# Why We Can’t Solve the Opioid Problem

**DOI:** 10.13023/jah.0103.05

**Published:** 2019-09-27

**Authors:** Wayne F. Coombs

**Affiliations:** None, wcoombs@marshall.edu

**Keywords:** Appalachia, biopsychosocial model, opioid crisis

## Abstract

Appalachia’s opioid epidemic is a complex, systemic problem being addressed by limited intervention processes conceptualized through narrow disciplinary models that are not working. We need a new comprehensive, collaborative approach if we ever hope to find solutions to this problem.

For the past several decades, there have been two primary ways to solve addiction problems…punishment and treatment. The devastating opioid crisis in Appalachia continues to grow, so it is clear that “treat them” or “lock them up” approaches are not working. Recently, Scutchfield^1^ acknowledged the importance of life saving therapies, but concludes, “this is not going to be enough,” a point on which we are in complete agreement.

So why can’t we solve this problem? The most significant barrier is the way we think about it, because this determines the solutions we develop. Our narrow conceptual thinking about the opioid problem is currently preventing us from finding what Scutchfield calls “the two major issues—etiology and intervention” that could put us on the path to solving the problem.

In a classic Indian parable, six blind men are sent to discover what an elephant is. They each touch the elephant in different parts and come to very different conclusions of what it is. They argue about who is right but end up being unable to agree. They are unable to grasp the full picture because of rigid adherence to their own points of view while ruling out other perspectives. In research, this is called conformation bias, which is the tendency to interpret information in a way that confirms one’s pre-existing beliefs.

The biomedical, psychological, and social sciences have all touched the opioid problem, but none has the full picture. Each one rules out the other and thus we have no etiology for the opioid problem or the other diseases of despair.^2^ I anticipate that this “blindness” will lead to increased competitiveness between the disciplines, which will grow more intense in the future given the likelihood of hundreds of millions or billions of dollars that will be coming into the system from legal settlements against the pharmaceutical industry.

There is no evidence that throwing boatloads of money at this problem in such a fragmented way will do anything to relieve our predicament. To uncover the etiology of the crisis and develop effective interventions, we are going to have to change our thinking. A much more comprehensive approach is needed, one that integrates multidisciplinary perspectives and knowledge into a new paradigm.

Fortunately, there is a way through the barrier. About 50 years ago, a physician named George Engel became exasperated with what he witnessed as dehumanizing medical care. He criticized the dualistic nature of the biomedical approach, which viewed not only the mind and body as separate, but also the body as the more “real” part of human life worthy of study. He rejected the notion of viewing the body as a machine and focusing only on the “diseases” while ignoring the people who suffered. He began seeing human well-being as a complex, interactive, connected system that could not be reduced to a single disease.

He published a paper^3^ proposing an alternative called the biopsychosocial model of health. It was a way to comprehensively understand the complex interactions of biological, psychological, and social factors that govern our lives. There has never been an attempt to understand Appalachia’s problems from this perspective. So, we are unable to uncover the etiology of the opioid crisis or to find more comprehensive, effective ways to solve these problems.

The opioid crisis along with the diseases of despair are complex problems with no simple solutions derived from narrow disciplinary models. Ackoff^4^ said, “Every problem interacts with other problems and is therefore part of a set of interrelated problems, a system of problems…I choose to call such a system a mess.” Rittel and Webber^5^ coined the term “wicked problems” for this phenomenon. The point is that complex problems require thinking that is capable of grasping the big picture, including the interrelationships among a full range of historical and underlying factors.

The biological, psychological, and social sciences do not work together, but instead pursue and preserve their own power and their status quo. We need a broader, more collaborative approach. We need a change to a biopsychosocial paradigm. If this can ever happen, it will depend on those who have the courage to try a new path and the wisdom to support it.
